# Epileptic discharges initiate from brain areas with elevated accumulation of α-amino-3-hydroxy-5-methyl-4-isoxazole propionic acid receptors

**DOI:** 10.1093/braincomms/fcac023

**Published:** 2022-02-07

**Authors:** Tomoyuki Miyazaki, Yutaro Takayama, Masaki Iwasaki, Mai Hatano, Waki Nakajima, Naoki Ikegaya, Tetsuya Yamamoto, Shohei Tsuchimoto, Hiroki Kato, Takuya Takahashi

**Affiliations:** 1 Department of Physiology, Yokohama City University Graduate School of Medicine, Yokohama 236-0004, Japan; 2 Department of Neurosurgery, National Center Hospital, National Center of Neurology and Psychiatry, Kodaira 187-8551, Japan; 3 Department of Neurosurgery, Yokohama City University Graduate School of Medicine, Yokohama 236-0004, Japan; 4 Division of System Neuroscience, National Institute for Physiological Sciences, Okazaki 444-8585, Japan; 5 Department of Nuclear Medicine and Tracer Kinetics, Osaka University Graduate School of Medicine, Suita 565-0871, Japan

**Keywords:** AMPA receptor, positron emission tomography, magnetoencephalography, intracranial electroencephalogram, equivalent current dipole

## Abstract

Presurgical identification of the epileptogenic zone is a critical determinant of seizure control following surgical resection in epilepsy. Excitatory glutamate α-amino-3-hydroxy-5-methyl-4-isoxazole propionic acid receptor is a major component of neurotransmission. Although elevated α-amino-3-hydroxy-5-methyl-4-isoxazole propionic acid receptor levels are observed in surgically resected brain areas of patients with epilepsy, it remains unclear whether increased α-amino-3-hydroxy-5-methyl-4-isoxazole propionic acid receptor-mediated currents initiate epileptic discharges. We have recently developed the first PET tracer for α-amino-3-hydroxy-5-methyl-4-isoxazole propionic acid receptor, [^11^C]K-2, to visualize and quantify the density of α-amino-3-hydroxy-5-methyl-4-isoxazole propionic acid receptors in living human brains. Here, we detected elevated [^11^C]K-2 uptake in the epileptogenic temporal lobe of patients with mesial temporal lobe epilepsy. Brain areas with high [^11^C]K-2 uptake are closely colocalized with the location of equivalent current dipoles estimated by magnetoencephalography or with seizure onset zones detected by intracranial electroencephalogram. These results suggest that epileptic discharges initiate from brain areas with increased α-amino-3-hydroxy-5-methyl-4-isoxazole propionic acid receptors, providing a biological basis for epileptic discharges and an additional non-invasive option to identify the epileptogenic zone in patients with mesial temporal lobe epilepsy.

## Teaser

Miyazaki *et al*. report that high [^11^C]K-2 uptake is observed closely to the location of equivalent current dipoles estimated by magnetoencephalography or seizure onset zones detected by intracranial EEG. These results suggest that epileptic discharges initiate from brain areas with increased AMPA receptors in patients with mesial temporal lobe epilepsy.

## Graphical Abstract

**Figure:**
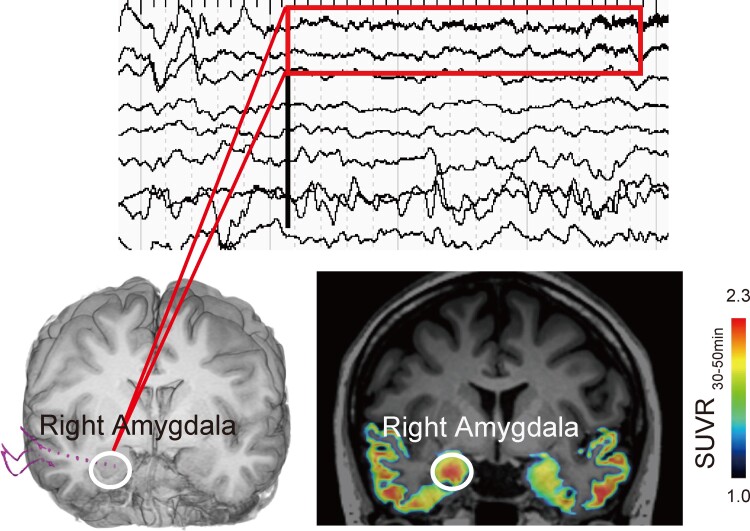

## Introduction

Approximately 20–30% of patients with epilepsy are drug-resistant and surgical removal of the clinically suspected epileptogenic zone is often effective for seizure control in patients with drug-resistant epilepsy.^1^ However, to improve surgical outcomes, there is a need for a diagnostic modality that can identify the entire epileptogenic zone directly.^2^ The glutamate α-amino-3-hydroxy-5-methyl-4-isoxazole propionic acid receptor (AMPAR) is a central mediator of neurotransmission in the brain.^3–12^ Surgically removed specimens from patients with epilepsy exhibit increased AMPARs.^13^ Furthermore, the administration of perampanel, a selective non-competitive AMPAR antagonist, reduces epileptic seizures.^14,15^ These results suggest that the elevation of AMPAR levels is related to epileptic activities. To visualize and quantify the density of AMPARs in living human brains, we recently developed the first PET tracer, [^11^C]K-2.^16–18^ Further, we found that [^11^C]K-2 represents the density of cell surface AMPARs.^17^ We detected elevated [^11^C]K-2 uptake in brain areas that included clinically identified epileptogenic zone.^16^ However, it remains unclear whether brain areas with high AMPAR accumulation initiate epileptic discharges in living human brains. Magnetoencephalography (MEG) signals are derived from the net effect of postsynaptic ionic currents that flow in the dendrites of neurons under synaptic transmission and are generated by a substantial population of neurons aligned in the same direction.^19,20^ Compared with EEG signals, MEG signals are less influenced by the difference in electrical conductivities between the CSF, skull and skin, as these tissues that surround the brain have constant magnetic permeability.^21^ Thus, MEG is a non-invasive modality that estimates the source of interictal epileptiform discharges (IEDs).^22^ Herein, IED was modelled using a single equivalent current dipole (ECD).^23^ Therefore, MEG can approximate the localization of IEDs and help identify the irritative zones. Removal of the ECD area is known to be associated with postoperative seizure control in patients with epilepsy.^24,25^ Here, we characterized the irritative and seizure onset zones (SOZs) using [^11^C]K-2 in combination with MEG or intracranial EEG (iEEG). We performed PET scans using [^11^C]K-2 and MEG in five patients with mesial temporal lobe epilepsy (MTLE) who underwent surgical resections of the suspected epileptogenic zones that were identified by the non-invasive evaluations. We found that ECDs colocalized with brain areas of elevated [^11^C]K-2 uptake in the surgically resected areas, compared with the contralateral hemisphere. Furthermore, we performed iEEG in one patient, in whom we detected higher [^11^C]K-2 uptake in the SOZ than in other brain areas. Thus, we demonstrated that elevated AMPAR levels underlie the genesis of epileptic discharges.

## Materials and methods

### Human participants

#### Patients with epilepsy

The clinical trial to evaluate the efficacy of [^11^C]K-2 in refractory epilepsy patients undergoing anterior temporal lobectomy (trial registry number UMIN000025090) was carried out to examine the efficacy of [^11^C]K-2 in measuring the density of AMPA receptors in patients with epilepsy. Detailed information about the participants and the study design was previously reported by Miyazaki *et al*.^16^ In short, eight patients diagnosed with refractory MTLE based on a non-invasive assessment met the eligibility criteria and were registered. Five of the eight patients underwent MEG, and one underwent iEEG, all of whom provided data that were analysed in this study. Five patients (one male and four females) with drug-resistant MTLE underwent respective temporal surgery at the National Center Hospital, National Center of Neurology and Psychiatry, and had undergone at least 1 year of postoperative follow-up. The clinical characteristics of the patients are summarized in Table 1. The mean age at the PET examination of [^11^C]K-2 was 39.8 years (range, 21–58 years). The mean duration from the diagnosis of epilepsy to the PET examination of [^11^C]K-2 was 32.4 years (range, 8–51 years).

**Table 1:** Clinical characteristics

Case	Sex	Age at the first seizure on life (Y)	Age at [^11^C]K-2-PET	AED at surgery (dose)	Seizure frequency	Location of MRI lesion	Location of scalp-EEG abnormality		Location of ECDs estimated by MEG (total number of ECDs)	Location of FDG-PET hypometablism	Location of [^11^C]K-2 high uptake	[^11^C]K-2 uptake in the hippocampus	Surgery	Pathological findings	Seizure outcome (ILAE classification)
							Interictal	Ictal							
1	F	4	27	CBZ(400) LEV(2000) LTG(175)	1–2/M	L hippocampus (HS)	L aT	L T	L anterior T (6)	L T (Diffuse)	L PHG	None	L ATL	HS	1
2	M	9	53	CBZ(800) PHT(250)	1–2/W	R hippocampus (HS)	R aT	R T	R Temporal pole and L mesial T (52)	R mesial T	R Temporal tip	None	R ATL	HS	1
3	F	4	40	LTG(300) CBZ(400)	1–2/W	L hippocampus (HS)	L aT	L T	L MTG and cortex facing bottom of occipitotemporal sulcus (2)	L T (Diffuse)	L PHG, FG, temporal tip	None	L ATL	HS	1
4	F	7	58	VPA(800) ZNS(300) CBZ(200) LTG(75) DZP(15)	1–3/W	R hippocampus (HS)	R a-mT	Non-localizable	R mesial and lateral T (32)	R T (Diffuse)	R MTG, ITG	None	R ATL	HS	1
5	F	13	21	LCM(400)	5/M	R Temporal tip (ambiguous)	R aT > L aT	R T	R and L T (18)	R T (Diffuse)	R Amygdala, hippocampus	High uptake	R ATL without hippocampectomy	Heterotopia	4

AED, anti-epileptic drug; aT, anterior temporal; a-mT, anterior-middle temporal; ATL, anterior temporal lobectomy; HS, hippocampal sclerosis; IFG, inferior frontal gyrus; L, left; MTG, middle temporal gyrus; R, right; T, temporal.

All five patients underwent presurgical comprehensive epilepsy evaluations that included medical interviews, neurological and neuropsychological examinations, long-term video-electroencephalography, MRI, ^18^F-fluorodeoxyglucose-PET (FDG-PET) and MEG. Epilepsy histories were obtained from the patients and their relatives, and the semiology of their habitual seizures was confirmed. The epilepsy diagnoses and classifications were established after comprehensive evaluations. The MRI evaluations revealed structural abnormalities in all patients, and four of the five had hippocampal sclerosis (Table 1). Abnormal hypometabolism on FDG-PET was also observed in all the patients.

### Magnetoencephalography

MEG was recorded with simultaneous scalp-EEG for 40-min periods during awake and sleep conditions using a 1000 Hz sampling rate and band-pass filter between 0.03 and 330 Hz, with a whole-head system that consisted of 204 planar gradiometers (Elekta, Helsinki, Finland). The location of the dipole sources that best fit the measured magnetic fields was calculated using a single ECD model at the peak of each spike. We selected and presented ECDs that showed >90% goodness-of-fit value for the evaluation. The estimated ECDs were plotted on the co-registered [^11^C]K-2 images using the PMOD Fusion tool v3.7 (PMOD Technologies). We qualitatively evaluated the locational relationship between the estimated ECDs and brain areas with high [^11^C]K-2 uptake.

### Intracranial electroencephalogram

EPI-5 underwent an intracranial electrode implantation. The EEG onset for each seizure was determined as the time at which unequivocal EEG changes appeared along with the seizure onset. Ictal EEG changes that started within 500 ms from the EEG onset were defined as early ictal iEEG changes.^26^ The region that corresponded to the electrode detecting early ictal iEEG changes was defined as the SOZ.

### *In vivo* PET and MRI imaging

[^11^C]K-2 was synthesized at Yokohama City University Hospital in accordance with the good manufacturing practice ordinance and was certified by the Japanese Society of Nuclear Medicine. PET imaging was performed with a Toshiba Aquiduo scanner (Toshiba Medical), which provided an axial field of view (FOV) of 240 mm and 80 contiguous 2.0-mm-thick slices. A 4.7-s transmission scan was performed for attenuation correction, and a 60-s intravenous injection of [^11^C]K-2 (378 ± 19 MBq) was administered, followed by an emission scan of 90 min, with frames of 12 × 10, 2 × 30, 7 × 60 s, 1 × 2, 1 × 3, 3 × 5 and 6 × 10 min. Dynamic images were reconstructed with a 2D-ordered subset expectation maximization using four iterations, 14 subsets, a 128 matrix, a zoom of 2.8 and a 5.0-mm Gaussian filter. To permit the accurate delineation of brain regions for data analysis, each participant underwent an MRI scan on a GE SIGNA 3.0-T scanner (GE Medical Systems). Images were acquired with a proton-density-weighted sequence [time to echo = 17 ms, repetition time = 6000 ms, FOV = 22 cm (two-dimensional), matrix = 256 × 256, slice thickness = 2 mm and number of excitations = 2].

### PET and MRI analysis

#### MRI segmentation

The T_1_-weighted MRI was segmented into probability maps of grey matter, white matter and CSF using the statistical parametric mapping (SPM)8 (Wellcome Department of Imaging Neuroscience). The grey matter and CSF probability maps were processed with an 8 mm full width half maximum Gaussian filter, to match the MRI spatial resolution with that of the PET scanner. The white matter areas were determined to fulfil the following conditions for voxel value: white matter probability map >0.9, smoothed grey matter map <0.05 and smoothed CSF map <0.05, according to a method described previously.^16^

#### Standardized uptake value ratio images

Since white matter is suitable as a reference region for [^11^C]K-2-PET images, no AMPA receptor expression was detected in the white matter, as described previously,^16^ we obtained standardized uptake value ratio (SUVR) images using white matter as a reference region. We found that the summation image of SUVR between 30 and 50 min (SUVR_30–50 min_) after radiotracer injection closely correlated with the AMPA receptors density.^16^ Furthermore, SUVR_30–50 min_−1 exhibited a good linear relationship with the non-displaceable binding potential [*BP*_ND_; a quantitative index of receptor density used in PET receptor imaging, quantified from the slope obtained from Logan graphical analysis (LGA) with white matter as a reference region],^27^ which showed that SUVR_30–50min_−1 is an appropriate surrogate outcome for *BP*_ND_.^16^ Based on these findings, the summation images of [^11^C]K-2 PET during 30–50 min after radiotracer injection were obtained from patients with MTLE and co-registered to the T_1_-weighted MRI. PET images and T_1_-weighted MRI images were spatially normalized into Montreal Numerological Institute (MNI) standard space using SPM12. To focus on temporal lobe imagings of [^11^C]K-2, the mask image of temporal lobe was applied, defined the following conditions for voxel value; grey matter map >0.5 and temporal lobe with Wake Forest University PickAtlas Tool. Finally, the masked PET images were transformed MNI space to native space using the inverse deformation field.

#### Quantitative analysis of [^11^C]K-2 uptake

To calculate SUVR values of arrow-indicated areas and contralateral areas at temporal lobe, the volume of interests (VOIs) were manually defined on the basis of the anatomically symmetrical coordinates. The VOIs consisted of 10 × 10 × 10 mm that were placed in fusiform gyrus (FG), middle temporal gyrus (MTG), hippocampus (HIP) and inferior temporal gyrus (ITG) using the PMOD Fusion tool v3.7 (PMOD Technologies). We calculated SUVR values and quantified values of the ratio of arrow-indicated areas to contralateral areas.

### Data availability

All requests for raw and analysed data are promptly reviewed by the Yokohama City University Research Promotion Department to determine whether the request is subject to any intellectual property or confidentiality obligations and, further, inspected by the Institutional Review Board of Yokohama City University Hospital. Upon these approvals, derived data will be released via a material transfer agreement from the corresponding author.

## Results

**Figure 1:**
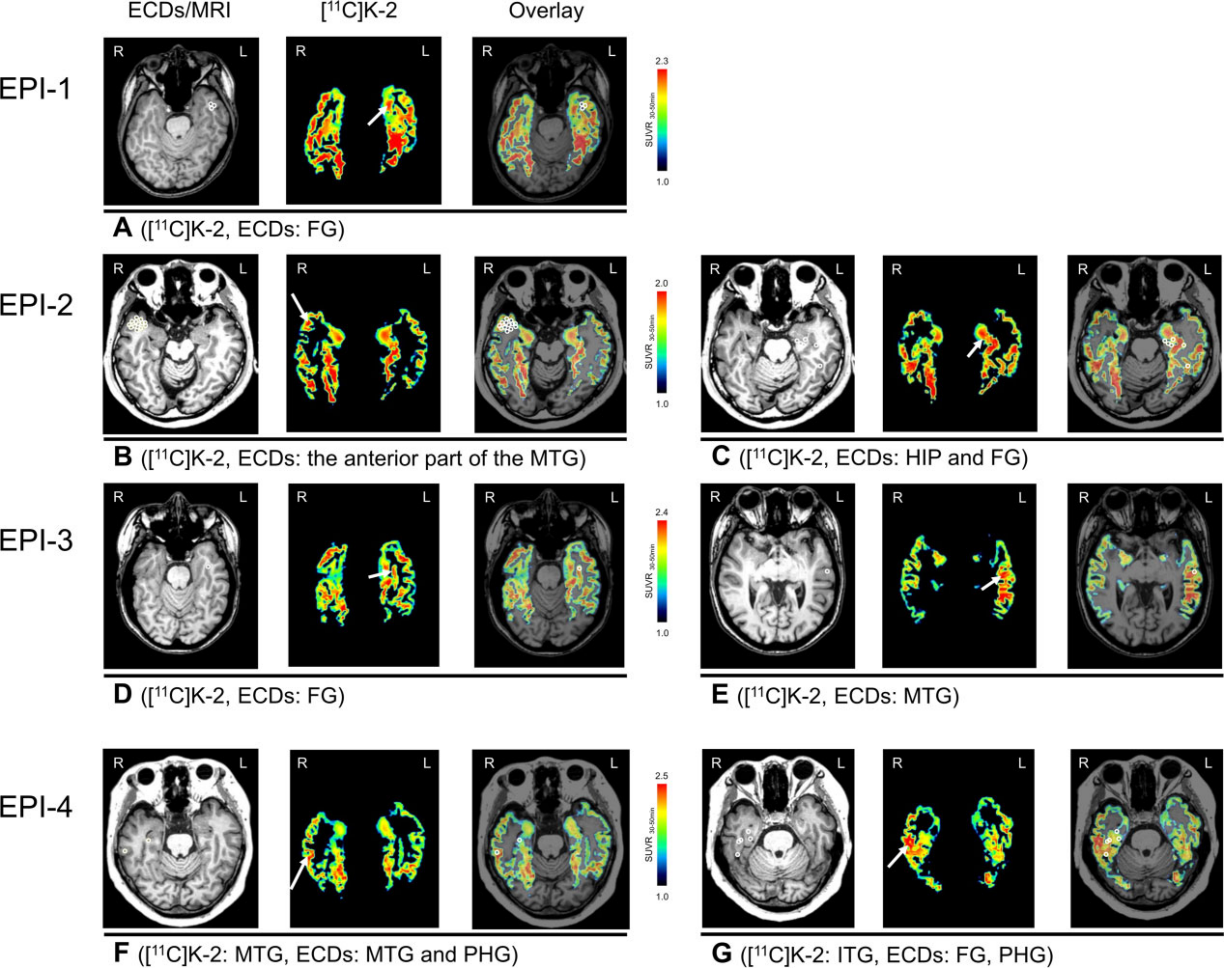
**Increased [^11^C]K-2 signals colocalize with ECDs in SUVR_30–50 min_ images of temporal lobes in patients with MTLE**. Representative [^11^C]K-2 SUVR_30–50 min_ images are shown restricted to temporal lobes where ECDs are estimated. Each panel shows ECDs on MRI (left), [^11^C]K-2 (middle) and overlay (right). White arrows indicate the area showing elevated [^11^C]K-2 uptakes and white circles indicate estimated ECDs. (**A**) EPI-1 showed elevated [^11^C]K-2 uptake and estimated ECDs in the left FG. EPI-2 showed elevated [^11^C]K-2 uptake and estimated ECDs in the anterior part of the right MTG (**B**) and, further, elevated [^11^C]K-2 uptake and estimated ECDs were identified in the left HIP and the anterior part of FG. One ECD (posterior white circle) was estimated in the posterior part of FG (**C**). EPI-3 showed elevated [^11^C]K-2 uptake (white arrow) and estimated ECD (white circle) in the left FG (**D**) and, further, elevated [^11^C]K-2 uptake and estimated ECDs were identified in left MTG (**E**). EPI-4 showed elevated [^11^C]K-2 uptake (white arrow) and estimated ECD (outer white circle) in MTG and the other ECD was estimated in the parahippocampal gyrus (PHG, inner white circle) (**F**). Furthermore, elevated [^11^C]K-2 uptake was identified in the right ITG and estimated ECDs were identified in FG, adjacent closely to ITG, PHG (**G**).

**Figure 2:**
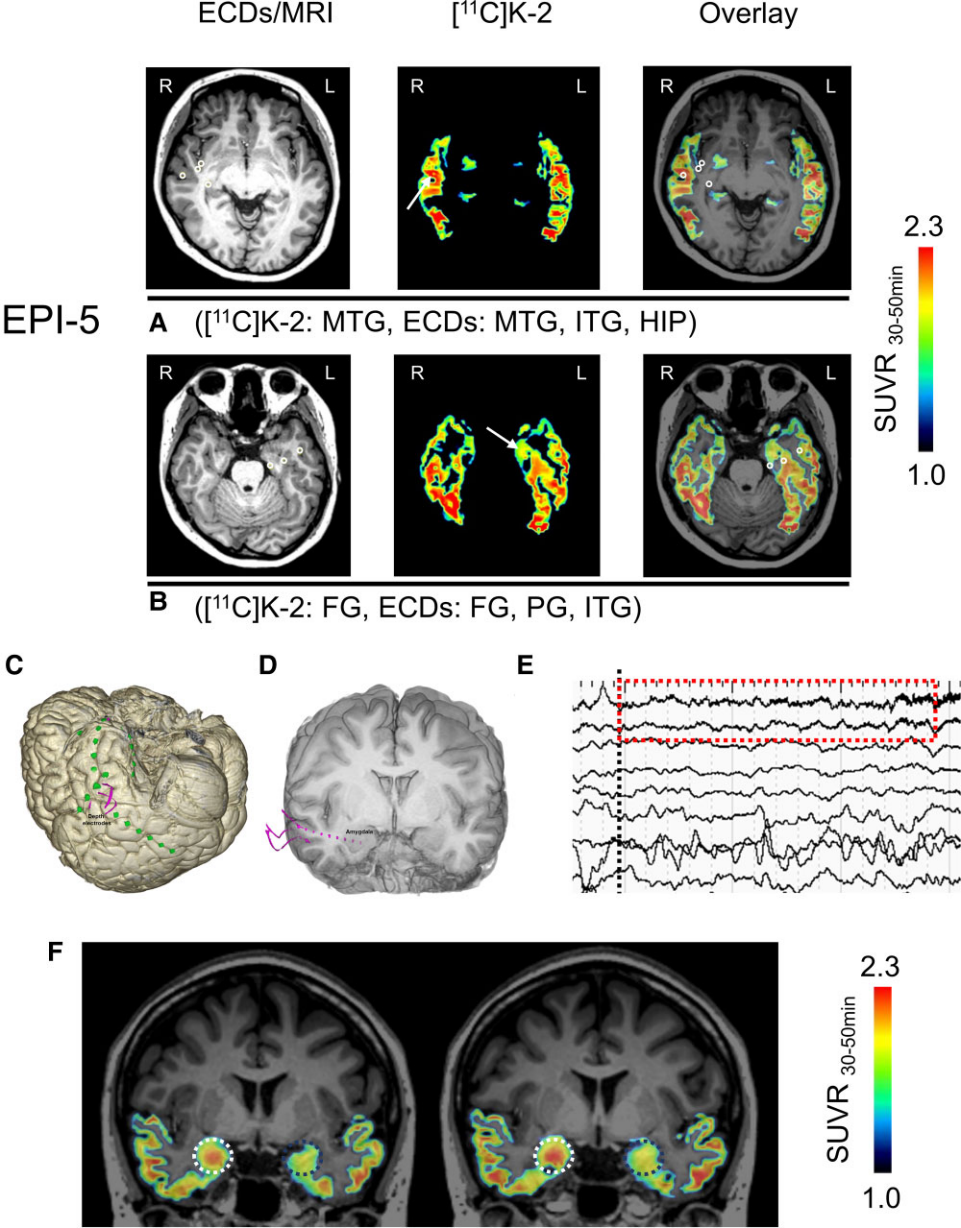
**[^11^C]K-2 is highly taken up in the SOZ**. (**A** and **B**) Representative [^11^C]K-2 SUVR_30–50 min_ images are shown, restricted to temporal lobes where ECDs are estimated. Each panel shows ECDs on MRI (left), [^11^C]K-2 (middle) and overlay (right). White arrows indicate the area showing elevated [^11^C]K-2 uptakes and white circles indicate estimated ECDs. EPI-5 showed elevated [^11^C]K-2 uptake and estimated ECDs in the right MTG and, further, other ECDs were estimated in right ITG and HIP (**A**). Furthermore, elevated [^11^C]K-2 uptake and estimated ECDs were identified in the left FG, and other ECDs were estimated in left PG and ITG (**B**). (**C**) The area where subdural electrodes are implanted in EPI-5. Pink lines show depth electrodes and green dots show brain surface electrodes. (**D**) Three depth electrodes were implanted targeting to right amygdala (pink lines and dots). (**E**) The iEEG change at the seizure onset (ictal EEG onset) is shown with black dots line. The earliest iEEG change (low-voltage fast activity indicated by red-dashed square) was detected by the depth electrode in right amygdala alone. The activity was the first unequivocal change at the ictal onset phase. Therefore, we deemed that the amygdala was the SOZ in this patient. (**F**) The zoomed image of red-dashed square in Fig. 2E shows that low-voltage fast activity occurs in the right amygdala. (**G**) SUVR_30–50 min_ images show that the elevated [^11^C]K-2 uptake colocalizes with the SOZ (right amygdala: white dots circle). The contralateral amygdala is indicated as a dotted blue circle.

To examine the role of AMPARs in the genesis of epileptic discharges, we PET-scanned five patients with MTLE who underwent anterior temporal lobectomy using [^11^C]K-2. First, we prepared the summation images of [^11^C]K-2 over 30–50 min after the radiotracer injection and created SUVR_30–50 min_ images using white matter as a reference region, as described previously.^16^ In the previous study, we demonstrated that SUVR_30–50 min_ of each VOI in the resected regions of these MTLE patients with the administration of [^11^C]K-2 represents the density of AMPARs.^16^ Further, LGA using white matter as a reference of these MTLE patients with the radiotracer injection exhibited linearity of the plot, indicating reversible binding kinetics of [^11^C]K-2 in these patients.^16^ From the slope of the plots obtained from LGA of these MTLE patients, *BP*_ND_ (a quantitative index of receptor density used in PET receptor imaging) was calculated, and the good linear relationship was observed between *BP*_ND_ and SUVR_30–50 min_, suggesting that SUVR_30–50 min_ can be an appropriate surrogate marker for *BP*_ND_.^16^ Thus, we used SUVR_30–50 min_ for the analysis of [^11^C]K-2 in MTLE patients described above. We also performed MEG in all five patients and iEEG in one patient (EPI-5). The locations of the ECDs that were estimated using MEG are described in Table 1, Figs 1 and 2A and B.

EPI-1 showed six ECDs in the left anterior part of the temporal lobe, five of which were estimated in the FG where we observed elevated [^11^C]K-2 uptake, compared with the contralateral hemisphere (Fig. 1A). EPI-2 showed 52 ECDs (41 in the right and 11 in the left temporal lobe), which mostly clustered in the right temporal pole that belongs to the anterior part of the superior and middle temporal gyri. This patient also showed increased [^11^C]K-2 uptake mainly in the right anterior part of the MTG of the temporal pole, with high colocalization of the right ECDs and increased [^11^C]K-2 (Fig. 1B). Eleven ECDs in the left temporal lobe were estimated in the HIP and FG, most of which were colocalized with increased [^11^C]K-2 (Fig. 1C). EPI-3 showed two ECDs, one in the left FG that faced the bottom of the occipitotemporal sulcus and the other in the left MTG. We found increased [^11^C]K-2 signals in these brain areas compared with the other hemispheres (Fig. 1D and E). EPI-4 showed 32 ECDs that were mostly distributed extensively in the right temporal lobe. We found colocalization of elevated [^11^C]K-2 uptake and ECDs in the lateral part of the right MTG (Fig. 1F). Furthermore, elevated [^11^C]K-2 uptake was observed in the right ITG, which was adjacent closely to fusiform and parahippocampal gyrus where some ECDs were estimated as shown in Fig. 1G. EPI-5 showed 18 ECDs in the bilateral temporal lobes sparsely (11 in the left temporal lobe and 7 in the right temporal lobe). In this patient, the ECDs were not clearly colocalized with increased [^11^C]K-2 signals in either temporal lobe (Fig. 2A and B). No ECDs were identified in the right amygdala, where we observed the highest [^11^C]K-2 uptake (Fig. 2F). Prior to surgical resection, the patient was implanted with iEEG electrodes (Fig. 2C and D). At the onset of the patient’s habitual seizures, low-voltage fast activities were detected on the depth electrode contacts implanted into the right amygdala (Fig. 2E and Supplementary Fig. 1), which was expected to be the SOZ, as well as increased [^11^C]K-2 uptake (Fig. 2F). These results suggest that epileptic discharges initiated from brain areas with elevated AMPAR levels.

To clarify the significant elevated [^11^C]K-2 uptake, we performed VOI-analysis by placing VOIs on the areas indicated by arrows in figures and VOIs on the contralateral areas to elucidate SUVR_30–50_ of each VOI (Table 2). All areas indicated by arrows in the figures had increased SUVR_30–50_ compared with those of contralateral sides with the statistical significance.

**Table 2:** Quantitative assessment of elevated [^11^C]K-2 uptake

Patient	Location	Figure panel	SUVR _30–50 min_		
			Arrow-indicated area	Contralateral area	Ratio
EPI-1	FG	Fig. 1A	2.05	1.78	15.12
EPI-2	Anterior MTG	Fig. 1B	1.83	1.64	11.22
	HIP and FG	Fig. 1C	1.81	1.76	3.30
EPI-3	FG	Fig. 1D	2.04	1.75	16.72
	MTG	Fig. 1E	2.35	2.23	5.05
EPI-4	MTG	Fig. 1F	2.40	2.04	17.56
	ITG	Fig. 1G	2.34	1.96	18.99
EPI-5	MTG	Fig. 2A	1.93	1.84	4.82
	FG	Fig. 2B	2.21	2.18	1.37
Average			2.10^a^	1.91	10.46
SD			0.21	0.19	6.49

^a^ Statistical significance between SUVRs of arrow-indicated area and contralateral area (*P* = 0.002, paired *t*-test, *n* = 9).

## Discussion

Although a number of previous studies have suggested a relationship between increased AMPARs and epileptic activities, it has not been clearly specified whether epileptic discharges originate from brain areas with high AMPAR expression in patients with epilepsy, due to the lack of technology to visualize AMPARs in living human brains. For example, surgical specimens were reported to contain increased AMPARs, and the strict brain areas that initiate epileptic discharge should be much smaller than the areas that undergo surgical resection.^28,29^ Furthermore, although the administration of perampanel, a non-competitive AMPAR antagonist, has been effective for refractory epilepsy,^15^ the anti-epileptic effect of systemic AMPAR antagonists administration does not prove that brain areas with elevated AMPAR expression are responsible for the initiation of epileptic discharges. In this study, using PET imaging with [^11^C]K-2, we found that epileptic discharges detected by MEG or iEEG initiated from brain areas with high AMPAR expression in patients with MTLE. Since AMPAR is a principal component of excitatory glutamatergic synaptic function, this study provides a strong biological basis for the genesis of epileptic discharges.

While MEG is a powerful non-invasive modality for localizing the epileptogenic zone, it has some technological and methodological limitations. First, MEG is far less sensitive to epileptic activities that occur in deep brain areas, such as the amygdala, HIP, insula and cingulum, in which MEG could not properly localize brain areas with epileptic discharges. In contrast, PET imaging with [^11^C]K-2 appeared to localize the SOZ in deep brain areas, as detected by iEEG. Second, MEG can miss rare and infrequent epileptic discharges due to the limited recording duration, while [^11^C]K-2 has the potential to identify the entire epileptogenic zone by measuring AMPAR expression levels. For these reasons, the areas with high uptake of [^11^C]K-2 are expected to be broader than the areas where ECDs are detected, leading to the mismatch of the areas indicated by [^11^C]K-2 and MEG. Indeed, increased [^11^C]K-2 uptakes were observed in the lateral temporal cortex especially in EPI-2 and 4. This observation does not contradict the diagnosis of MTLE, since some degree of epileptogenicity exists in the lateral temporal cortex. These two patients had the long history of epilepsy, which facilitated the establishment of the ‘epileptic’ neural networks to connect the mesial and lateral temporal regions.^30^ IEDs have often been observed in the lateral temporal cortex beyond the mesial temporal structures and sometimes beyond the surgical resection areas, and these propagations were correlated with unfavourable seizure outcomes after surgery.^31^ Furthermore, the cumulative evidence indicated that the selective treatment to the HIP or to the mesial temporal structures were inferior to the anterior temporal lobectomy in terms of the chance of seizure freedom.^32,33^ The finding of the elevated uptake of [^11^C]K-2 in the lateral temporal cortex might be useful for the prediction of treatment outcomes.

On the other hand, patients other than EPI-5 did not show increased [^11^C]K-2 uptake in the affected HIP even though these patients had hippocampal sclerosis that has been considered to be epileptogenic zone as an aetiology of epilepsy (Table 1). As a reason for this mismatch, we expect that EPI 1–4 had reduced number of hippocampal neurons as their pathological findings, and elevated uptakes of [^11^C]K-2 were not observed in the affected HIP even though the number of AMPA receptor in a living single neuron might be increased that could result in epileptic discharges. This expectation suggests that limited number of neurons having increased AMPA receptors is sufficient to introduce clinical seizure. It is also plausible that homeostatic plasticity reverts the expression of AMPA receptor in the affected HIP and mesial temporal cortex where the ECDs were not accompanied by the high uptake of [^11^C]K-2 observed in EPI-2 and 4. Previous works supported this hypothesis that the increased AMPA receptors in the epileptogenic zones may gradually decrease to normal levels potentially due to the synaptic scaling.^34–36^ This hypothesis might propose the unique characteristic of [^11^C]K-2 that could delineate the currently active regions where epileptic discharges are observed frequently and, in the other words, homeostatic plasticity has not been achieved.

Thus, PET imaging with [^11^C]K-2 provides a powerful non-invasive tool for identifying the potential epileptogenic zone, thereby increasing diagnostic accuracy during presurgical evaluations and eventually improving surgical outcomes. Clinically, the introduction of PET imaging with [^11^C]K-2 can dramatically change the presurgical practice of refractory epilepsy.

## Ethics statement

This study was carried out as a retrospective secondary analysis using a dataset that was obtained from the clinical study; UMIN000025090 for the clinical trial with patients with epilepsy (the date of the first treatment was March 27, 2017, and that of the last treatment was January 7, 2019). The secondary analysis was performed under protocols approved by the Yokohama City University Human Investigation Committee in accordance with the ethical guidelines for medical and health research involving human subjects drawn up by the Japan Ministry of Health, Labor and Welfare. The secondary analysis protocol was registered under the number jRCTs1030210154.

## Supplementary Material

fcac023_Supplementary_Data

## Abbreviations

ECD =: equivalent current dipole
FDG =: ^18^F-fluorodeoxyglucose
FG =: fusiform gyrus
FOV =: field of view
HIP =: hippocampus
IED =: interictal epileptiform discharge
ITG =: inferior temporal gyrus
LGA =: Logan graphical analysis
MEG =: magnetoencephalography
MNI =: Montreal Numerological Institute
MTG =: middle temporal gyrus
MTLE =: mesial temporal lobe epilepsy
SOZ =: seizure onset zone
SPM =: statistical parametric mapping
SUVR =: standardized uptake value ratio
VOI =: volume of interest
