# Supplementary figures and images for: Epileptic discharges initiate from brain areas with elevated accumulation of α-amino-3-hydroxy-5-methyl-4-isoxazole propionic acid receptors
Source: Brain Commun. 2022 Feb 7;4(2):fcac023. doi: 10.1093/braincomms/fcac023 (PMC8994107; doi:10.1093/braincomms/fcac023)

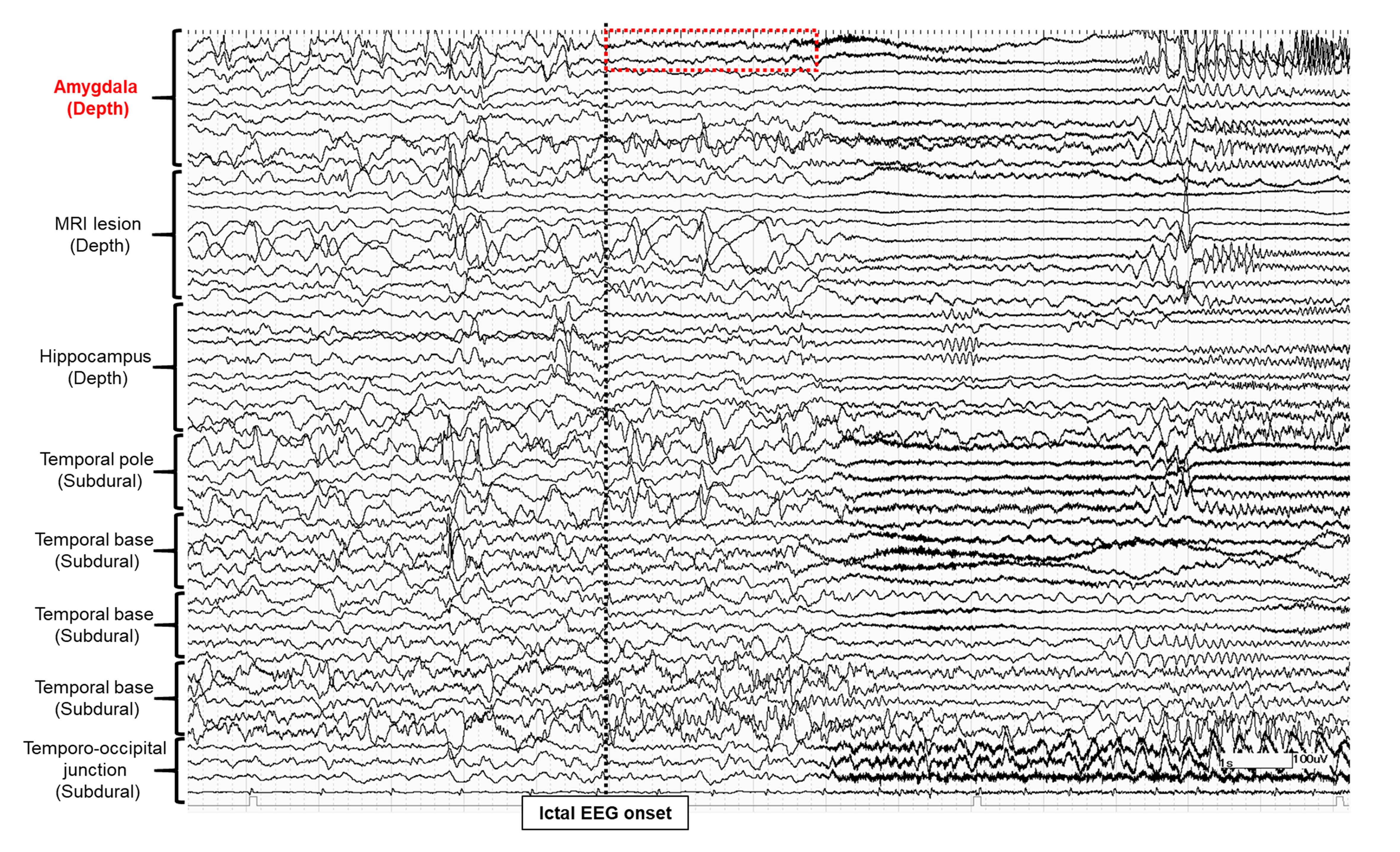

Supplement: fcac023_Supplementary_Data [file fcac023_supplementary_data.jpeg]
